# Data from the MOSAiC Arctic Ocean drift experiment

**DOI:** 10.1038/s41597-022-01678-8

**Published:** 2022-09-15

**Authors:** Stephan Frickenhaus, Daniela Ransby, Matthew Shupe, Ralf Jaiser, Marcel Nicolaus

**Affiliations:** 1 Alfred Wegener Institute Helmholtz Centre for Polar and Marine Research, Bremerhaven, Germany; 2 University Bremen, Centre for Industrial Mathematics, Bremen, Germany; 3 University of Colorado, CIRES and NOAA, Colorado, USA; 4 Alfred Wegener Institute Helmholtz Centre for Polar and Marine Research, Potsdam, Germany

**Keywords:** Atmospheric dynamics, Databases, Carbon cycle, Biogeochemistry, Physical oceanography

## Abstract

The Multidisciplinary drifting Observatory for the Study of Arctic Climate (MOSAiC) is a multinational interdisciplinary endeavor of a large earth system sciences community.

MOSAiC^1^ resulted in an unprecedented amount and diversity of observation data over the central Arctic sea ice (Fig. 1): over 660 unique sensors or measurement devices with >8200 registered events, resulting in more than 90000 managed parameters in 73 endorsed sub-projects have been recorded (see https://mosaic-expedition.org/). 122 PIs are registered to work on the data. The year-round experiment started in September 2019 -and ended in October 2020 after a long-lasting preparation phase. The Implementation planning meeting in November 2017 in St. Petersburg was the starting point of its data logistics and data management concepts, with its core organizational component being the MOSAiC Data Policy^2^, described further below. As technical core component the MOSAiC Central Storage (MCS) was drafted, to be operated on board of the research icebreaker Polarstern^3^ and mirrored on land, leg by leg, allowing for data sharing and collaboration amongst researchers of the MOSAiC consortium.

**Fig. 1:**
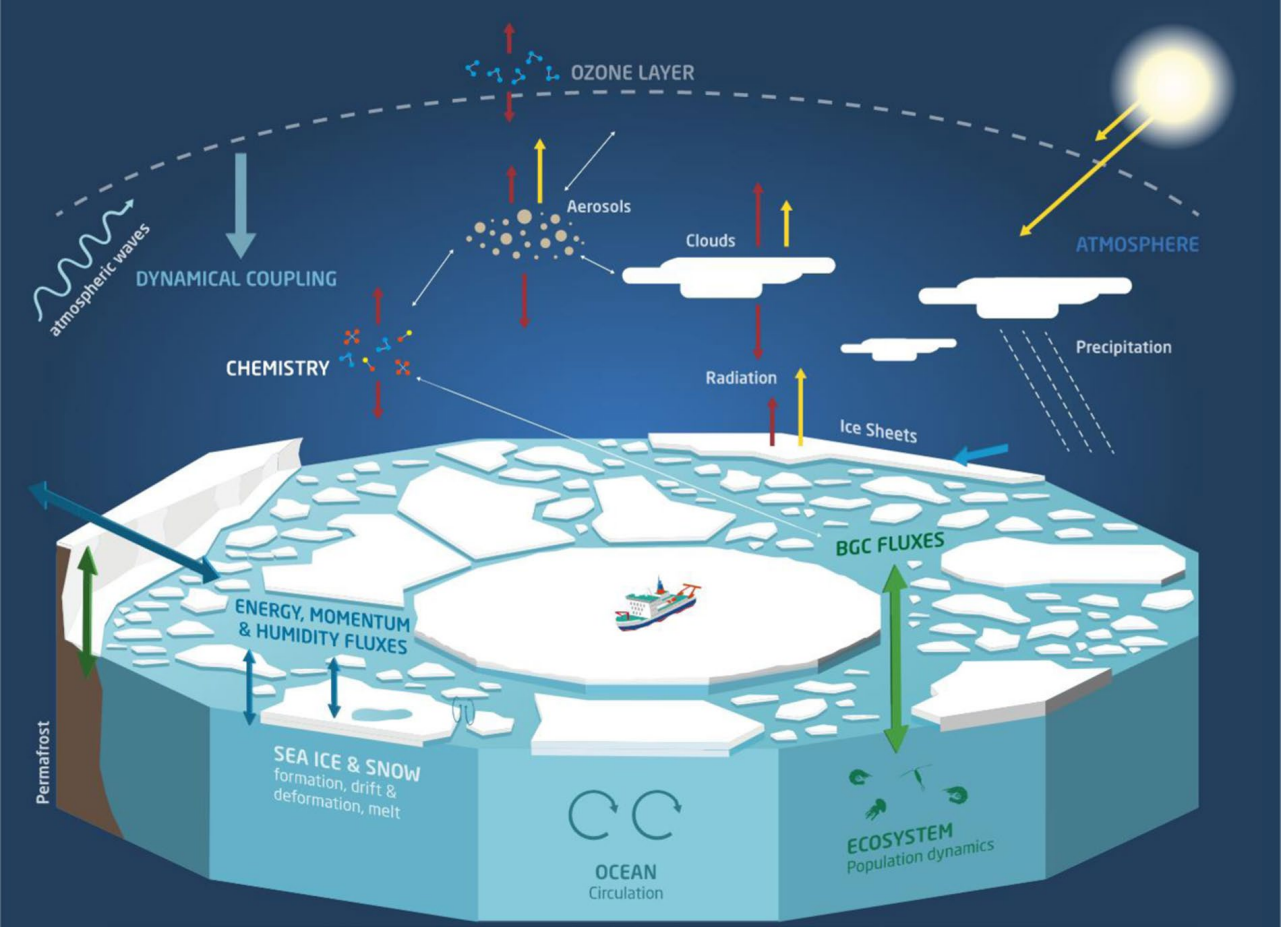
Illustration of the coupled Arctic System, as it was observed during the MOSAiC 2019/2020 expedition (C) Alfred Wegener Institute/eventfive.

Another milestone was the Science Meeting in May 2018 in Potsdam, where the term "MOSAiC Data Legacy" has been coined, to pronounce the importance of unique data management principles, technologies, and joint publication strategies in this multi-national consortium. In this aspect, the SHEBA experiment from the late 90ies was seen as a reference^4^. SHEBA Phase II was a year-long experiment taking place from October 1997 to October 1998 in the Arctic Ocean. It generated published data that is the basis of a series of scientific papers (see https://www.eol.ucar.edu/node/644/publications). The SHEBA data legacy has been taken up by the Arctic Data Centre, a long-term data repository, in the form of 184 individually citable datasets (https://arcticdata.io/catalog/data).

The MOSAiC Data Policy^2^ is the central document defining membership of scientists and data users in the MOSAiC Consortium, and collaboration principles in terms of data storage, data provisioning, data sharing and data publication. Furthermore, it defines the latest date for MOSAiC consortium members to make their data publicly available as 31 January 2023. The overall aim is a rapid dissemination of the scientific data to a diverse community of stakeholders to enable scientific studies of the rapidly changing Arctic system. The consortium includes major data centers and repositories as Pangaea and the Arctic Data Centre. The MOSAiC project stands out for its enormous heterogeneity of data sets, including in-situ observations and samples, sensor data, remote sensing and airborne data, and numerical model results (see process picture Fig. 1). All data are published under the FAIR principles.

A first series of three overview papers from the MOSAIC science teams focused on Sea Ice/Snow, Physical Oceanography, and Atmosphere were published in the journal Elementa^5–7^. Additional scientific overviews are forthcoming.

The following collection provides descriptions of primary and derived datasets from MOSAiC. Comments on data management and international collaboration with data repositories will be included, describing the complexity and heterogeneity of data sets. The collection will be extended by more data descriptors and comments, and authors are welcome to participate or initiate this kind of publication. A collection website will give further information on ongoing MOSAiC activities, such as conferences and publications (https://www.nature.com/collections/dcihcgabdc).
